# Pathological T-staging in patients undergoing total laryngectomy with prior tracheostomy: A retrospective study

**DOI:** 10.1016/j.bjorl.2026.101907

**Published:** 2026-09-22

**Authors:** Thomas Peter Maahs, Maurício Fontoura Ferrão, Nathália Perusso Becker, Renan Vieira de Brito, Carlos Takahiro Chone, Nédio Steffen, Gerson Schulz Maahs

**Affiliations:** a Pontifícia Universidade Católica do Rio Grande do Sul (PUCRS), Hospital São Lucas, Departamento de Otorrinolaringologia e Cirurgia de Cabeça e Pescoço, Porto Alegre, RS, Brazil; b Universidade Estadual de Campinas (UNICAMP), Departamento de Otorrinolaringologia e Cirurgia de Cabeça e Pescoço, Campinas, SP, Brazil; c Hospital de Clínicas de Porto Alegre, Departamento de Otorrinolaringologia e Cirurgia de Cabeça e Pescoço, Porto Alegre, RS, Brazil

**Keywords:** Laryngectomy, Neoplasm staging, Tracheostomy, Laryngeal neoplasms, Squamous cel carcinoma

## Abstract

**Objective:**

The 2018 American Joint Committee on Cancer (AJCC) classification for laryngeal cancer defines staging based on anatomical invasion but does not formally incorporate prior tracheostomy. Discrepancies between clinical staging (cT) and pathological staging (pT) often complicate therapeutic decision-making. The objective of this study was to determine if the clinical necessity of a prior tracheostomy serves as an independent preoperative predictor of pT4a staging in patients undergoing primary total laryngectomy, thereby assisting in clinical planning.

**Methods:**

This retrospective study reviewed the histopathological records of patients surgically treated for advanced laryngeal squamous cell carcinoma over a 20-year period. Eligibility criteria required a preoperative clinical staging indicative of extensive disease justifying total laryngectomy, without the presence of prior chemoradiotherapy or partial laryngeal surgeries. Data regarding pathological staging, nodal involvement, and microscopic characteristics were extracted. Associations were assessed using Fisher's Exact Test, and the magnitude of effect was calculated using multivariate logistic regression, yielding an Odds Ratio (OR).

**Results:**

The final cohort comprised 96-patients (93.75% male; median age 65-years). A prior tracheostomy was required in 39.6% of the cohort. A statistically significant association was established between pT4a staging and prior tracheostomy, with 86.8% of tracheostomized patients revealing pT4a disease on final pathology (p = 0.013). Multivariate logistic regression confirmed that advanced pathological staging (T4a) was the sole independent predictor of the need for prior tracheostomy (OR = 4.41, 95% CI; p = 0.010). Other variables, including nodal staging, histological grade, perineural invasion, and angiolymphatic invasion, did not show significant independent associations with the requirement for prior tracheostomy.

**Conclusion:**

The clinical requirement of a tracheostomy prior to definitive total laryngectomy is a robust, independent predictor of pT4a staging. In the context of ambiguous clinical imaging, prior tracheostomy serves as a surrogate marker for extensive anatomical compromise, providing critical adjunctive data to support multidisciplinary tumor boards in decisions favoring primary surgical resection.

**Level of evidence:**

3.

## Highlights

• Preoperative tracheostomy is strongly linked to advanced tumor staging (pT4a).
• Microscopic traits do not independently predict the need for prior tracheostomy.
• Tumor extension predicts tracheostomy better than microscopic aggressiveness.

## Introduction

Accurate local tumor staging in laryngeal squamous cell carcinoma is paramount for defining curative therapeutic strategies. The 2018 American Joint Committee on Cancer (AJCC) TNM classification defines locally advanced glottic and supraglottic tumors (T3 and T4) based on precise anatomical parameters, including laryngeal immobility, thyroid and cricoid cartilage invasion, pre-epiglottic space involvement, and extralaryngeal extension.^1^ Current therapeutic mainstays for these locally advanced stages rely on either primary surgery (total laryngectomy), often followed by adjuvant therapy, or organ-preservation strategies utilizing exclusive radiotherapy or concurrent chemoradiotherapy.^2^, ^3^, ^4^

Historically, patients with extensive disease staged as T3 and T4a were managed uniformly with total laryngectomy.^2^^,^^5^ Beginning in the 1990s, there was a profound paradigm shift toward treating patients with locally advanced disease using chemoradiotherapy.^6^^,^^18^ However, long-term outcome analyses evaluating these shifts have demonstrated that patients with highly advanced T4a tumors specifically experience superior survival rates when treated primarily with total laryngectomy.^7^^,^^8^ Consequently, accurately distinguishing T3 from T4a disease preoperatively is a critical determinant of patient survival and therapeutic success.^19^

However, clinical staging (cT) heavily relies on videolaryngoscopy and cross-sectional imaging, which possess inherent limitations.^9^ Clinical imaging frequently struggles to differentiate peritumoral inflammation and edema from true neoplastic cartilage invasion, leading to well-documented discrepancies between clinical and final pathological staging (pT).^10^^,^^11^ While the AJCC staging system meticulously catalogs anatomical extent, it does not formally account for physiological events such as the necessity of a prior tracheostomy.^1^ Clinically, an emergency or planned tracheostomy prior to definitive treatment is a recognized physiological marker of severe laryngeal dysfunction, airway compromise, and high tumor burden.^12^

A critical gap in the current literature is the lack of robust histopathological correlation determining whether the physiological necessity of a tracheostomy can reliably predict the true pathological stage of the tumor.^11^^,^^12^ The objective of this study was to analyze the postoperative histological staging (pT) of patients with advanced laryngeal squamous cell carcinoma, determining whether prior tracheostomy functions as an independent predictor of pT4a disease to aid in therapeutic decision-making.

## Methods

### Study design and ethical considerations

This retrospective, cross-sectional cohort study utilized a long-term institutional clinical database. The protocol was formally approved by the PUCRS under Opinion No. 5.173.259, strictly adhering to the ethical principles of the Declaration of Helsinki (World Medical Association). Patient identity and confidentiality were rigorously maintained throughout data extraction and analysis, with data identified solely by randomized project registration numbers.

### Participants and eligibility criteria

The study comprehensively reviewed the histopathological examinations of paraffin-embedded surgical specimens from individuals with laryngeal squamous cel carcinoma who underwent primary total laryngectomy over a 20-year period. Inclusion criteria required a preoperative clinical intent for total laryngectomy based on advanced clinical staging (cT3 or cT4a). To ensure a homogeneous cohort relying solely on the primary biological behavior of the tumor, stringent exclusion criteria were applied: 1) Previous clinical treatments (absence of prior chemotherapy and/or radiotherapy) to eliminate the severe confounding effects of treatment-induced tumor downstaging and fibrosis; 2) Prior partial laryngeal surgeries; 3) primary tumors originating in the hypopharynx, given their fundamentally distinct lymphatic drainage patterns and worse prognostic behavior; 4) T4b tumors, due to their unresectable nature involving prevertebral fascia or major vessels; and 5) Histopathological diagnoses other than squamous cel carcinoma.

### Variables

The primary dependent variable was the performance of a tracheostomy prior to the definitive total laryngectomy. Independent variables systematically extracted from the final histopathological reports included pathological tumor staging (pT), pathological nodal staging (pN: negative vs. positive), histological grade of differentiation (low grade ‒ we l and moderately differentiated ‒ vs. high grade ‒ poorly differentiated), perineural invasion (present vs. absent), and angiolymphatic invasion (present vs. absent). Standard demographic variables (age and sex) were also recorded.

### Statistical analysis

Data were systematically organized and descriptively analyzed using frequency tables, cross-tabulations (crosstabs), and percentages. Bivariate associations between the categorical clinicopathological variables and the performance of prior tracheostomy were assessed utilizing Fisher’s Exact Test. To identify independent predictors and determine the magnitude of the effect, a multivariate analysis was conducted using a logistic regression model, generating Odds Ratios (OR). Variables exhibiting perfect separation (sex and age) were excluded from the multivariate logistic model to preserve statistical validity. The final multivariate model included T-staging (T4a vs. T2–T3), N staging, degree of differentiation, perineural invasion, and angiolymphatic invasion. A l statistical tests were two-tailed, with results considered statistically significant at an alpha level of p ≤ 0.05.

## Results

The final study sample comprised 96 individuals who met all rigorous eligibility criteria. The cohort demonstrated a stark male predominance, with 90 males (93.75%) and 6 females (6.25%). The age range spanned from a minimum of 28-years to a maximum of 87-years, presenting a median age of 65-years (Table 1). A tracheostomy was required prior to the total laryngectomy in 38 individuals (39.6%), whereas 58 patients (60.4%) were managed surgically without the need for a prior surgical airway (Table 2).

**Table 1:** Descriptive statistics for the age variable.

Variable	Minimum	Maximum	Average	Standard Deviation
Age (years)	28.0	87.0	65.4	10.7

**Table 2:** Description of study variables.

Variable	Reply	Nº of cases	%
Prior Tracheostomy	No	58	60.4
	Yes	38	39.6
Staging	T2	4	4.2
	T3	24	25.0
	T4a	68	70.8
Staging N	Negative	70	72.9
	Positive	26	27.1
Age Group	Under 70	25	26.0
	70‒80	41	42.7
	But of 70	30	31.3
Sex	Women	6	6.3
	Male	90	93.8
Degree of Differentiation	Well differentiated	7	7.3
	Moderately Differentiated	70	72.9
	Little Differentiated	19	19.8
Perineural Invasion	No	70	72.9
	Yes	26	27.1
Angiolymphatic Invasion	No	64	66.7
	Yes	32	33.3

Following comprehensive postoperative histopathological analysis, the true pathological staging (pT) of the sample was distributed as follows: 4 patients (4.2%) were classified as pT2, 24 (25%) as pT3, and 68 (70.8%) as pT4a (Table 2). The presence of pT2 cases within a cohort preoperatively scheduled for total laryngectomy represents instances of clinical-to-pathological staging discrepancy, where initial clinical assessments (cT3/cT4a) overestimated the true microscopic disease extent.

Bivariate analysis (Table 3) evaluated the cross-referencing between main clinical and pathological variables and prior tracheostomy. A statistically significant association was observed between advanced staging and prior tracheostomy (p = 0.013). Strikingly, patients harboring advanced disease (T4a) presented a significantly higher frequency of tracheostomy, with 86.8% of the tracheostomized cohort confirmed as pT4a, compared to minimal frequencies in the T3 (10.5%) and T2 (2.6%) subgroups.

**Table 3:** Crossovers with previous tracheostomy.

		Prior Tracheostomy				
		No		Yes		p
Variable	Reply	n	%	n	%	
Staging	T2	3	5.2%	1	2.6%	0.013^a^
	T3	**20**	**3**4.5%	4	10.5%	
	T4a	35	60.3%	**33**	**8**6.8%	
Staging N	Negative	41	58.6%	29	41.4%	0.641NS
	Positive	17	65.4%	9	34.6%	
Age Group	Under 70	14	24.1%	11	28.9%	0.462NS
	70–80	23	39.7%	18	47.4%	
	But of 70	21	36.2%	9	23.7%	
Sex	Women	**6**	**1**0.3%	‒	‒	0.044^a^
	Male	52	89.7%	**38**	**10**0.0%	
Degree of Differentiation	Well differentiated	3	5.2%	4	10.5%	0.218NS
	Moderately Differentiated	46	79.3%	24	63.2%	
	Little Differentiated	9	15.5%	10	26.3%	
Perineural Invasion	No	41	70.7%	29	76.3%	0.358NS
	Yes	17	29.3%	9	23.7%	
Angiolymphatic Invasion	No	42	72.4%	22	57.9%	0.105NS
	Yes	16	27.6%	16	42.1%	

NS, Not Significant.

a Significant p ≤ 0.05.

A statistically significant association was also found between sex and tracheostomy (p = 0.044), noting that no women in the sample required prior tracheostomy. However, this specific result should be interpreted with caution, as it is intrinsically related to the small number of females in the sample and the higher general epidemiological prevalence of laryngeal tumors in males. The distribution by age group did not demonstrate any significant association with prior tracheostomy (p = 0.462).

Crucially, microscopic indicators of tumor aggressiveness failed to show significant correlations. There was no statistically significant association between nodal staging (N negative vs. positive) and the presence of prior tracheostomy (p = 0.641). Histological differentiation grade showed no significant association (p = 0.218). The presence of perineural invasion was not associated with a higher frequency of prior tracheostomy (p = 0.358). Similarly, there was no significant association between angiolymphatic invasion and prior tracheostomy (p = 0.105), despite a higher numerical trend in the group with invasion (42.1% vs. 27.6%).

In the multivariate analysis via logistic regression (Table 4 and Fig. 1), advanced pathological staging (T4a) emerged as the sole independent and statistically significant predictor associated with a higher probability of prior tracheostomy, generating an Odds Ratio of 4.41 (p = 0.009). The remaining variables ‒ positive N staging, poorly differentiated grade, perineural invasion, and angiolymphatic invasion ‒ did not demonstrate any independent association after statistical adjustment.

**Table 4:** Multivariate analysis by logistic regression.

Variable	Coefficient (β)	OR	p-value	95% IC
Advanced staging (T4a)	1.48	4.41	0.010	1.44–13.58
Positive N Estacy	−0.37	0.69	0.49	0.24–1.97
Poorly differentiated degree	0.58	1.78	0.310	0.58–5.45
Perineural invasion	–0.96	0.38	0.104	0.12–1.22
Angiolymphatic invasion	0.80	2.22	0.146	0.736–6.49

**Fig. 1:**
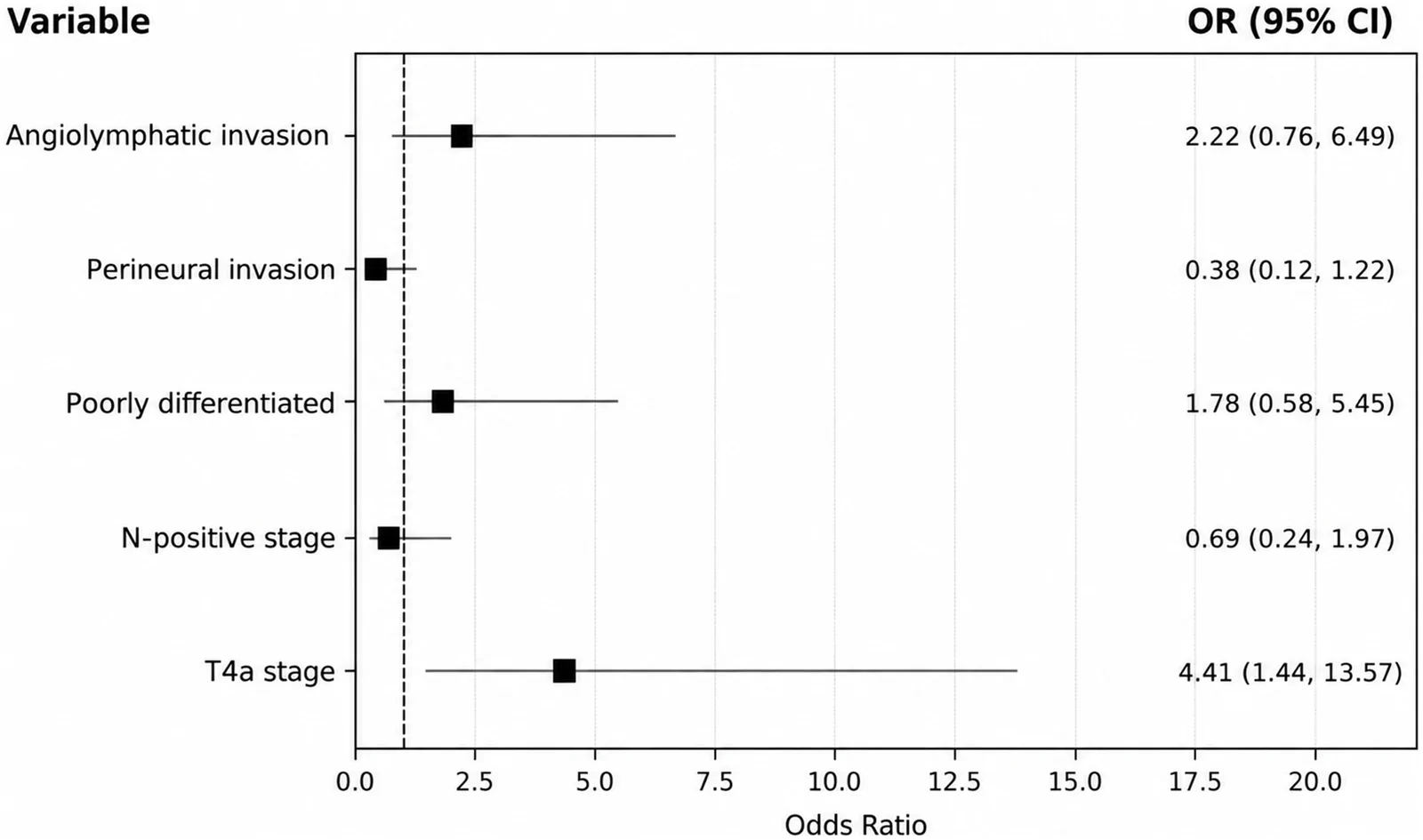
Multivariate analysis by logistic regression – Forest Plot Model.

## Discussion

### Literature gap and study rationale

The decision to pursue larynx-preservation strategies utilizing chemoradiation versus primary total laryngectomy remains one of the most consequential debates in head and neck oncology.^2^, ^3^, ^4^ The AJCC 2018 guidelines effectively stratify tumor risk based on strict anatomical boundaries.^1^ However, these anatomical guidelines omit profound physiological markers of disease burden, specifically the necessity of a preoperative tracheostomy. While the prognostic impact of tracheostomy has been historically questioned regarding stomal recurrence,^13^ there is a pervasive lack of studies directly correlating the clinical physiological need for an airway intervention with discrepancies between clinical and true pathological staging. This study was explicitly designed to fill this literature gap, evaluating whether prior tracheostomy can serve as a dependable clinical surrogate for pT4a disease.

### Key findings and clinical message

The paramount finding of this study is that advanced pathological staging (pT4a) serves as the single independent predictor of the need for a prior tracheostomy (OR = 4.41; p = 0.009). Nearly 87% of patients requiring a surgical airway harbored pT4a tumors. This observation carries immense practical weight for clinical decision-making. Because clinical staging via cross-sectional imaging can be highly ambiguous ‒ often failing to definitively confirm or rule out true thyroid or cricoid cartilage invasion^10^ ‒ the physiological event of airway obstruction necessitating a tracheostomy act as a decisive “red flag”. It unequivocally indicates that anatomical tumor extension and direct airway compromise are the primary drivers of the disease state. In multidisciplinary tumor boards, an ambiguous cT3/cT4a scan coupled with a tracheostomy should strongly direct the clinical consensus toward primary surgical resection.

### Secondary findings and histopathological context

Our multivariate data further illuminated that microscopic indicators of tumor aggressiveness, such as perineural and angiolymphatic invasion, did not correlate with the necessity for tracheostomy. This supports the mechanical and volumetric, rather than purely microscopic, nature of airway obstruction in these patients. Additionally, the identification of four pT2 patients within a cohort preoperatively scheduled for total laryngectomy highlights the pervasive clinical issue of pathological downstaging.^10^^,^^11^ Inflammatory processes and localized edema can easily mimic cT3/cT4a disease on preoperative imaging. Notably, none of these up-staged pT2 patients required a prior tracheostomy, which inversely validates our thesis: an intact airway strongly correlates with less extensive true cartilaginous involvement, despite misleading imaging.

### Representativeness of the study population

The demographic profile of the analyzed sample is highly representative of the broader epidemiological landscape of laryngeal squamous cell carcinoma. The pronounced male predominance (93.75%) aligns precisely with global epidemiological estimates, including the National Cancer Institute (INCA) registry data,^15^^,^^16^ which consistently document significantly higher incidence rates among males due to cumulative behavioral risk factors such as tobacco and alcohol consumption.

### External validity and comparison with prior literature

Our findings strongly resonate with modern surgical oncology literature which advocates for primary surgery in highly advanced cases. Chen and Halpern,^7^ as well as Grover et al.,^8^ have definitively established that T4a tumors specifically achieve superior overall survival outcomes when treated with total laryngectomy rather than chemoradiation. Furthermore, our data aligns seamlessly with the subset observations of Chu et al.,^17^ where 81% of laryngeal cancer patients presenting with severe airway obstruction harbored pT4 disease. Conversely, studies evaluating organ preservation protocols frequently observe high failure rates in patients with severe initial airway compromise.^2^^,^^4^^,^^14^ By demonstrating mathematically that tracheostomy predicts pT4a (OR = 4.41), our data provides actionable evidence supporting the historical assertions of Carrillo et al.,^11^ justifying surgical treatment which is oncologically better established in these advanced cases.

### Criticism of methods

The study utilized remarkably stringent inclusion criteria by analyzing exclusively patients who were naive to radiotherapy or chemotherapy. While this approach represents a significant methodological strength ‒ as it completely eliminates the profound confounding effects of radiation-induced tumor shrinkage, necrosis, and fibrosis on pathological staging ‒ it inherently reduced the available sample size. Furthermore, analyzing a retrospective dataset spanning 20-years introduces the variable of technological and diagnostic evolution. The resolution of preoperative CT scans and radiologic interpretation paradigms have advanced significantly over two decades, which may have influenced the accuracy of clinical staging and the threshold for early surgical intervention in earlier cases.

### Limitations of the study

The primary limitations of this study encompass its retrospective, non-randomized design and a relatively small sample size (n = 96). This limited sample is largely a consequence of total laryngectomy currently serving as a late-line surgical treatment or being strictly reserved for advanced neoplasms. Crucially, because the study focused strictly on the histopathological correlation between preoperative physiological events (tracheostomy) and precise staging (pT), comprehensive long-term longitudinal oncological outcomes ‒ such as stomal recurrence rates, disease-free survival, and long-term overall survival ‒ were not systematically analyzed or reported. Future multi-institutional prospective studies utilizing larger cohorts are necessary to link this predictive staging correlation with long-term survival metrics across diverse clinical scenarios.

## Conclusions

The clinical requirement for a tracheostomy prior to definitive total laryngectomy is strongly and significantly associated with pT4a staging, acting as an independent predictor of advanced extralaryngeal or cartilaginous disease. Microscopic variables, such as perineural or angiolymphatic invasion, do not independently drive the need for airway intervention. In clinical scenarios where preoperative imaging yields ambiguous or borderline staging between T3 and T4a, a prior tracheostomy serves as a highly reliable clinical surrogate marker for definitive pT4a disease. These findings provide critical support for multidisciplinary clinical decision-making, emphasizing that tracheostomized patients with advanced laryngeal cancer may be optimaly managed with primary surgical resection rather than organ-preservation protocols.

## ORCID ID

Nédio Steffen: 0000-0001-7892-6629

## Funding

There is no financial or material supports.

## Data availability

The authors declare that all data are available in repository.

## Declaration of competing interest

The authors declare no conflicts of interest.
